# Dynamic time warp analysis of individual symptom trajectories in patients with bipolar disorder

**DOI:** 10.1192/j.eurpsy.2023.1209

**Published:** 2023-07-19

**Authors:** R. Mesbah, M. Koenders, A. T. Spijker, M. de Leeuw, A. M. van Hemert, E. J. Giltay

**Affiliations:** 1 Psychiatry, Leiden University Medical Centre (LUMC), Leiden; 2 3Mental Health Care PsyQ Kralingen, Department of Mood Disorders, Rotterdam; 3 Faculty of Social Sciences, Institute of Psychology; 4 Mental Health Care Rivierduinen, Outpatient Clinic; 5 Mental Health Care Rivierduinen, Bipolar Disorder Outpatient Clinic, Leiden, Netherlands

## Abstract

**Introduction:**

Manic and depressive mood states in bipolar disorder (BD) may emerge from the non-linear relations between constantly changing mood symptoms exhibited as a complex dynamic system. Dynamic Time Warp (DTW) is an algorithm that may capture symptom interactions from panel data with sparse observations over time.

**Objectives:**

The current study is the first to analyze a time series of depression and manic symptoms using DTW analyses in patients with BD. We studied interactions and relative changes in symptom severity within and between participants.

**Methods:**

The Young Mania Rating Scale and Quick Inventory of Depressive Symptomatology were repeatedly assessed in 141 patients with BD, with on average 5.5 assessments per patient every 3 to 6 months. DTW calculated the distance between each of the 27*27 pairs of standardized symptom scores. The changing profile of standardized symptom scores of BD patients was analyzed in individual patients, yielding symptom dimensions in aggregated group-level analyses. Using an asymmetric time-window, symptom changes that preceded other symptom changes (i.e., Granger causality) yielded a directed network.

**Results:**

The mean age of the patients was 40.1 (SD 13.5) years old, and 60% were female. Idiographic symptom networks were highly variable between patients. Yet, nomothetic analyses showed five symptom dimensions: core (hypo)mania (6 items), dysphoric mania (5 items), lethargy (7 items), somatic/suicidality (6 items), and sleep (3 items). Symptoms of the ‘Lethargy’ dimension showed the highest out-strength, and its changes preceded those of ‘somatic/suicidality’, while changes in ‘core (hypo)mania’ preceded those of ‘dysphoric mania’.

**Image:**

**Figure:**
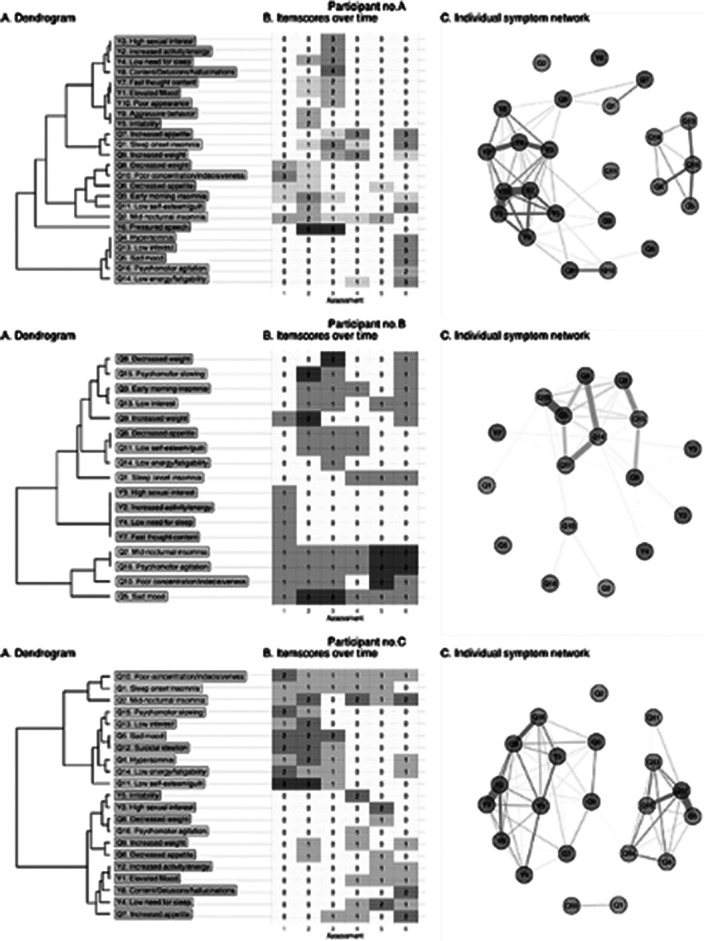

**Image 2:**

**Figure:**
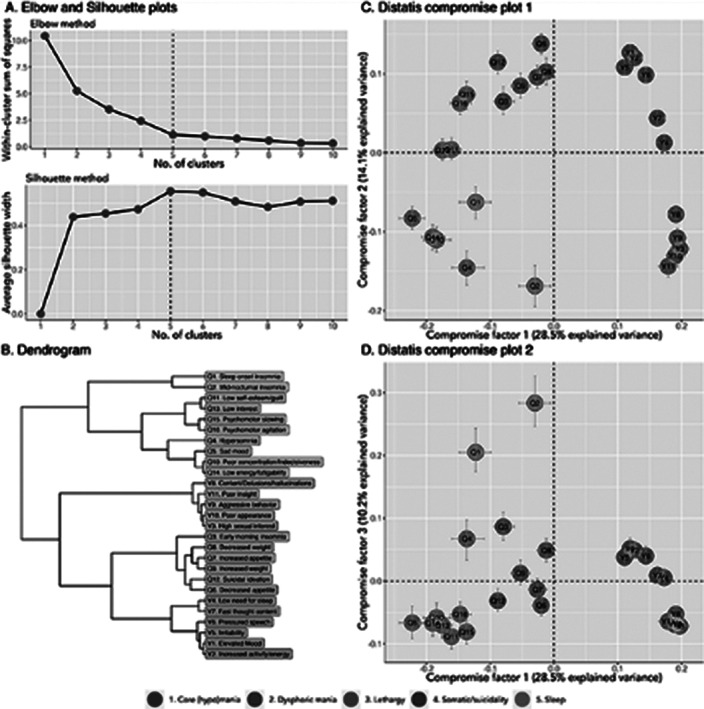

**Image 3:**

**Figure:**
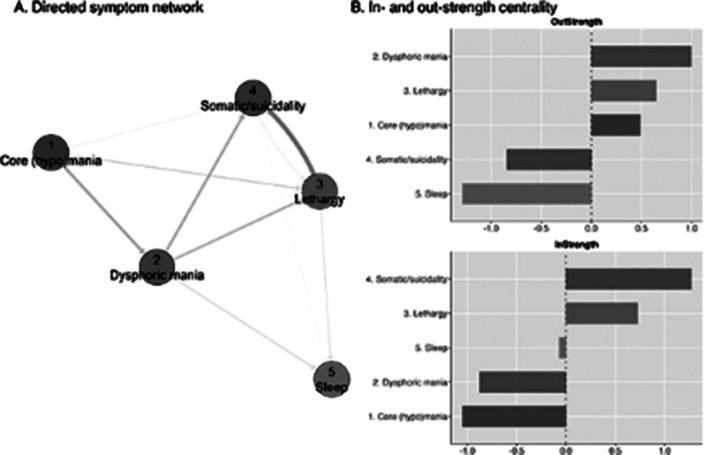

**Conclusions:**

DTW may help to capture meaningful BD symptom interactions from panel data with sparse observations. It may increase insight into the temporal dynamics of symptoms, as those with high out-strength (rather than high in-strength) could be promising targets for intervention.

**Disclosure of Interest:**

None Declared

